# Bi@Sn Core–Shell Structure with Compressive Strain Boosts the Electroreduction of CO_2_ into Formic Acid

**DOI:** 10.1002/advs.201902989

**Published:** 2020-10-01

**Authors:** Yulin Xing, Xiangdong Kong, Xu Guo, Yan Liu, Qiuyao Li, Yuzhe Zhang, Yelin Sheng, Xupeng Yang, Zhigang Geng, Jie Zeng

**Affiliations:** ^1^ Hefei National Laboratory for Physical Sciences at the Microscale CAS Key Laboratory of Strongly‐Coupled Quantum Matter Physics Key Laboratory of Surface and Interface Chemistry and Energy Catalysis of Anhui Higher Education Institutes Department of Chemical Physics University of Science and Technology of China Hefei Anhui 230026 P. R. China

**Keywords:** Bi@Sn catalysts, CO_2_ electroreduction, compressive strain, core–shell structure

## Abstract

As a profitable product from CO_2_ electroreduction, HCOOH holds economic viability only when the selectivity is higher than 90% with current density (*j*) over −200.0 mA cm^−2^. Herein, Bi@Sn core–shell nanoparticles (Bi core and Sn shell, denoted as Bi@Sn NPs) are developed to boost the activity and selectivity of CO_2_ electroreduction into HCOOH. In an H‐cell system with 0.5 m KHCO_3_ as electrolyte, Bi@Sn NPs exhibit a Faradaic efficiency for HCOOH (FE_HCOOH_) of 91% with partial *j* for HCOOH (*j*_HCOOH_) of −31.0 mA cm^−2^ at −1.1 V versus reversible hydrogen electrode. The potential application of Bi@Sn NPs is testified via chronopotentiometric measurements in the flow‐cell system with 2.0 m KHCO_3_ electrolyte. Under this circumstance, Bi@Sn NPs achieve an FE_HCOOH_ of 92% with an energy efficiency of 56% at steady‐state *j* of −250.0 mA cm^−2^. Theoretical studies indicate that the energy barrier of the potential‐limiting step for the formation of HCOOH is decreased owing to the compressive strain in the Sn shell, resulting in the enhanced catalytic performance.

## Graphical abstract

Bi@Sn core–shell nanoparticles (Bi core and Sn shell) are developed to boost the activity and selectivity of CO_2_ electroreduction into HCOOH. The lattice mismatch between the Bi core and Sn shell generates a compressive strain in the Sn shell. The compressive strain in the Sn shell decreases the energy barrier for the formation of HCOOH, resulting in the enhanced catalytic performance.

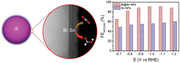

The excessive utilization of fossil fuels and accelerating emissions of CO_2_ have led to the energy shortage and greenhouse effect.^[^ ^1^, ^2^, ^3^, ^4^, ^5^ ^]^ CO_2_ electroreduction into useful chemicals and fuels represents a promising way that not only meets the ever‐increasing energy demands but also mitigates environmental crisis caused by CO_2_ emissions.^[^ ^6^, ^7^, ^8^, ^9^, ^10^, ^11^ ^]^ As a value‐added product from CO_2_ electroreduction, formic acid (HCOOH) is an important feedstock for pharmaceutical and chemical industry.^[^ ^12^ ^]^ Meanwhile, HCOOH is a liquid fuel for proton‐exchange membrane fuel cell.^[^ ^13^ ^]^ Additionally, HCOOH also serves as potential hydrogen carrier.^[^ ^14^ ^]^ Based on the gross‐margin model, HCOOH has been suggested to be one of the most economically viable products during CO_2_ electroreduction process.^[^ ^15^ ^]^ To this end, the electroreduction of CO_2_ into HCOOH is of great significance.

Currently, various metal‐based electrocatalysts such as Pd, Pb, Hg, Cd, Tl, In, and Sn have been explored to achieve the high activity and selectivity for electroreduction of CO_2_ into HCOOH.^[^ ^16^, ^17^, ^18^, ^19^, ^20^, ^21^, ^22^ ^]^ Among these catalysts, Sn‐based catalysts have drawn considerable attentions due to the superiorities of nontoxicity, earth abundance, and low cost.^[^ ^23^, ^24^, ^25^, ^26^ ^]^ Up to now, several effective strategies have been exploited to improve the catalytic performance of Sn‐based catalysts. For instance, owing to the abundant grain boundaries, the ultrathin sub‐2 nm SnO_2_ quantum wires composed by individual SnO_2_ quantum dots achieved improved Faradaic efficiency for HCOOH (FE_HCOOH_) of 87.3% with the current density (*j*) of −15.7 mA cm^−2^ relative to SnO_2_ nanoparticles (NPs).^[^ ^27^ ^]^ Besides, Sn quantum sheets confined in graphene exhibited high conductivity and fast charge‐transfer process, resulting in improved catalytic activity for HCOOH.^[^ ^28^ ^]^ Moreover, the mesoporous SnO_2_ displayed a maximum FE_HCOOH_ of 75% and a *j* of −10.8 mA cm^−2^ at −1.15 V versus reversible hydrogen electrode (vs RHE).^[^ ^29^ ^]^ The enhanced catalytic performance was attributed to the promoted CO_2_ activation by the construction of oxygen vacancy.^[^ ^29^ ^]^ However, most previously reported Sn‐based catalysts still suffer from limited FE_HCOOH_ at high current density, prohibiting the practical application of Sn‐based electrocatalysts. Therefore, it is of great importance to develop efficient Sn‐based catalysts with high activity and selectivity for HCOOH toward CO_2_ electroreduction.

Herein, we developed Bi@Sn core–shell nanoparticles (Bi core and Sn shell, denoted as Bi@Sn NPs) to boost the activity and selectivity for electroreduction of CO_2_ into HCOOH. In H‐cell system with 0.5 m KHCO_3_ as electrolyte, Bi@Sn NPs exhibited an FE_HCOOH_ of 91% with partial *j* for HCOOH (*j*_HCOOH_) of −31.0 mA cm^−2^ at −1.1 V versus RHE. The potential application of Bi@Sn NPs was testified via chronopotentiometric measurements in flow‐cell system with 2.0 m KHCO_3_ electrolyte. Under this circumstance, Bi@Sn NPs achieved an FE_HCOOH_ of 92% with a steady‐state *j* of −250.0 mA cm^−2^. Theoretical studies indicate that energy barrier of the potential‐limiting step for the formation of HCOOH was decreased owing to the compressive strain in Sn shell, resulting in the enhanced catalytic performance.

Bi@Sn NPs were synthesized via electroreduction of Bi_2_Sn_2_O_7_ NPs in 0.5 m KHCO_3_ at −0.8 V versus RHE for 1 h. Specifically, Bi_2_Sn_2_O_7_ NPs were prepared via a solvothermal reaction (Figure S1, Supporting Information). **Figure** **1**a shows the transmission electron microscopy (TEM) image of Bi@Sn NPs, which took a spherical morphology with an average diameter of 45 nm. As shown in Figure 1b, the high‐resolution TEM (HRTEM) image of an individual Bi@Sn NP exhibited a clear contrast between the Bi core and the Sn shell, indicating the core–shell structure of Bi@Sn NPs. To further characterize the exquisite core–shell structure of Bi@Sn NPs, we employed the high‐angle annular dark field scanning TEM (HAADF‐STEM). As shown in Figure 1c, the interplanar spacing for Sn (101) and (020) planes in Sn shell was 0.270 and 0.283 nm, respectively, less than the standard values of 0.279 and 0.291 nm in tetragonal Sn. Other regions of the Bi@Sn NP were also analyzed via HAADF‐STEM. As shown in Figure S2 in the Supporting Information, the HAADF‐STEM images of different regions exhibited the similar feature to that in Figure 1c. As shown in Figure S3 in the Supporting Information, the total distance of five groups of successive (020) plane was measured and then divided by five to obtain the interplanar spacing of (020) plane. The interplanar spacing of (020) plane for Sn shell in Bi@Sn NP was 0.283 nm. For comparison, the interplanar spacing of (020) plane for Sn NP was determined to be 0.291 nm, slightly larger than that of Sn shell in Bi@Sn NP. These results suggested that the lattice of Sn shell was compressed. In the Bi core, the lattice fringe with an interplanar spacing of 0.323 nm was ascribed to the (012) plane of rhombohedral Bi. Meanwhile, the thickness of Sn shells in Bi@Sn NPs ranged from 2.160 to 2.700 nm, revealing that the shell was composed of 8–10 layers of Sn atoms. The core–shell structure of Bi@Sn NPs was further confirmed by the energy dispersive X‐ray (EDX) elemental mapping. As shown in Figure 1g, the interior core of Bi (green) was surrounded by the outer shell of Sn (red). This result was also supported by the line‐scanning profiles across an individual Bi@Sn NP (Figure 1h). To investigate the phase composition of Bi@Sn NPs, we carried out X‐ray diffraction (XRD) measurement. As evidenced by XRD patterns in Figure 1i, the Bi@Sn NPs exhibited the diffraction peaks at 30.74°, 32.09°, and 44.98°, which were indexed to the (200), (101), and (211) planes of tetragonal Sn (JCPDS No. 89‐2761).^[^ ^5^ ^]^ The diffraction peaks of Sn (200) and Sn (101) in Bi@Sn NPs shifted to higher diffraction angles, further proving that the lattice of Sn shell was compressed (inset of Figure 1i; Table S1, Supporting Information). In addition, the diffraction peaks at 22.56°, 27.22°, 38.02°, 39.71°, 48.80°, 56.20°, 62.38°, and 64.69° were assigned to the (003), (012), (104), (110), (202), (024), (116), and (122) planes of rhombohedral Bi (JCPDS No. 85‐1330).^[^ ^30^ ^]^ Bi@Sn NPs exhibited a larger *I*_Bi(012)_/*I*_Bi(104)_ value (4.18) than that (2.95) of standard rhombohedral Bi, suggesting that the Bi core exhibited preferred orientations of (012) facets. The strong diffraction peak located at 26.48° was attributed to the (002) plane of graphite due to the substrate of carbon paper. These results together revealed that Bi@Sn NPs consisted of metallic Sn and Bi. Cyclic voltammogram (CV) measurements confirmed that Bi core was totally covered by Sn shell (Figure S4, Supporting Information). For comparison, we also prepared Sn NPs with an average diameter of 48 nm by reducing SnCl_2_ with NaBH_4_ (Figure S5, Supporting Information).

**Figure 1:**
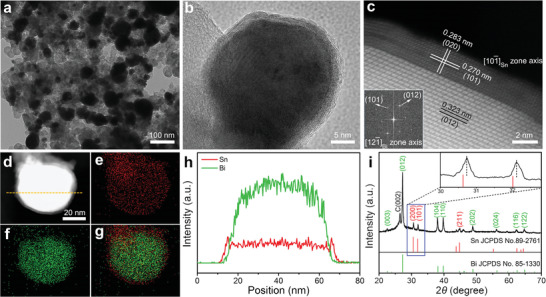
a) TEM image, b) HRTEM image, and c) HAADF‐STEM image of Bi@Sn NPs. d–g) HAADF‐STEM and EDX elemental mapping images of an individual Bi@Sn NP. h) Line‐scanning profiles of Sn and Bi along the yellow line in (d). i) XRD pattern of Bi@Sn NPs; the corresponding magnified XRD pattern of Bi@Sn NPs is given in the inset.

Bi@Sn NPs were applied as a heterogeneous catalyst toward CO_2_ electroreduction in an H‐cell system. Linear sweep voltammetry (LSV) curves of Bi@Sn NPs were measured in both CO_2_‐saturated and Ar‐saturated 0.5 m KHCO_3_ electrolytes. As shown in Figure S6 in the Supporting Information, Bi@Sn NPs exhibited a *j* of 45.1 mA cm^−2^ in CO_2_‐saturated electrolyte at −1.2 V versus RHE, which was 1.6 times as high as that (27.2 mA cm^−2^) in Ar‐saturated electrolyte. In this regard, CO_2_ electroreduction was more favorable than competing hydrogen evolution reaction (HER) over Bi@Sn NPs. In addition, based on the intercept of the linear region in Tafel plots, the exchange current density (*j*_0_) for HER over Bi@Sn NPs was calculated to be 16.1 µA cm^−2^, which was lower than that (35.4 µA cm^−2^) over Sn NPs (Figure S7, Supporting Information). In this case, Bi@Sn NPs restrained the competing HER relative to Sn NPs. We applied in situ attenuated total reflection infrared (ATR‐IR) spectroscopy to monitor the process of CO_2_ electroreduction over Bi@Sn NPs at different potentials in CO_2_‐saturated 0.5 m KHCO_3_ electrolyte (Figure S8, Supporting Information). The characteristic band at 1372 cm^−1^ for the symmetric O–C–O stretching mode of HCOOH was observed, indicating the formation of HCOOH over Bi@Sn NPs.^[^ ^31^, ^32^ ^]^

To evaluate the catalytic performance of Bi@Sn NPs and Sn NPs toward CO_2_ electroreduction, we conducted chronoamperometry measurements with a series of applied potentials. The gaseous products and liquid products were quantitatively analyzed via online gas chromatography and ^1^H nuclear magnetic resonance (^1^H NMR), respectively (Figure S9, Supporting Information). **Figure** **2**a shows the FE_HCOOH_ toward CO_2_ electroreduction over Bi@Sn NPs and Sn NPs. The FE_HCOOH_ of Bi@Sn NPs was higher than that of Sn NPs at all applied potentials. Specifically, at −1.1 V versus RHE, the FE_HCOOH_ of Bi@Sn NPs was 91%, which was 1.6 times as high as that (56%) of Sn NPs. Meanwhile, Bi@Sn NPs exhibited lower Faradaic efficiency for CO (FE_CO_) and Faradaic efficiency for H_2_ (FE_H2_) than Sn NPs at all applied potentials, demonstrating that Bi@Sn NPs suppressed the formation of CO and H_2_ (Figure S10, Supporting Information). As shown in Figure 2b, the *j*_HCOOH_ of Bi@Sn NPs was higher than that of Sn NPs at all applied potentials. Especially, when the applied potential was set at −1.2 V versus RHE, the *j*_HCOOH_ of Bi@Sn NPs reached −38.0 mA cm^−2^, whereas the *j*_HCOOH_ of Sn NPs was −20.9 mA cm^−2^. Figure S11 in the Supporting Information shows the yield rates for HCOOH of Bi@Sn NPs and Sn NPs at different applied potentials. At −1.2 V versus RHE, the yield rate for HCOOH of Bi@Sn NPs achieved 708.9 µmol cm^−2^ h^−1^, which was 1.8 times as high as that (390.6 µmol cm^−2^ h^−1^) of Sn NPs.

**Figure 2:**
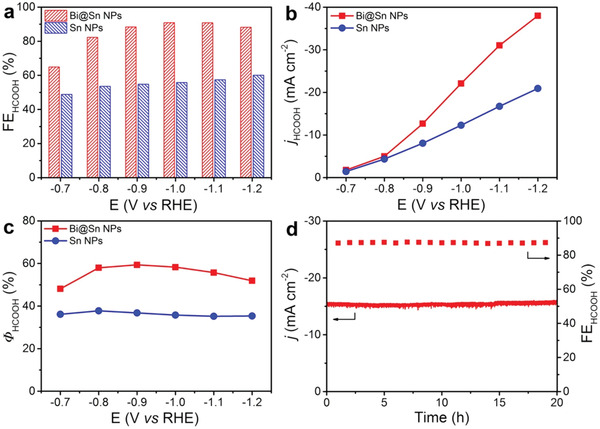
a) FE_HCOOH_, b) *j*_HCOOH_, and c) *Φ*_HCOOH_ of Bi@Sn NPs and Sn NPs at different applied potentials. d) The *j* and FE_HCOOH_ of Bi@Sn NPs at −0.9 V versus RHE with 20 h potentiostatic test.

The efficiency from electrical energy to the chemical energy of target product is also an important parameter to evaluate the catalytic performance.^[^ ^27^, ^33^ ^]^ Figure 2c shows the energy efficiency for HCOOH (*Φ*_HCOOH_) of Bi@Sn NPs and Sn NPs at different applied potentials. The *Φ*_HCOOH_ of Bi@Sn NPs exceeded 50% in a wide potential range from −0.8 to −1.2 V versus RHE. Notably, at −0.9 V versus RHE, the *Φ*_HCOOH_ of Bi@Sn NPs achieved 59%, which was 1.6 times as high as that (36%) of Sn NPs. The catalytic stability for CO_2_ electroreduction over Bi@Sn NPs was also evaluated via the chronoamperometry electrolysis. As shown in Figure 2d, Bi@Sn NPs exhibited negligible decay in both FE_HCOOH_ and *j* at −0.9 V versus RHE during 20 h electrolysis. The morphology, core–shell structure, and phase for Bi@Sn NPs were still preserved after the durability test (Figure S12, Supporting Information).

To rationalize the enhanced activity for HCOOH of Bi@Sn NPs relative to that of Sn NPs, we conducted the electrochemical surface areas (ECSAs) and electrochemical impedance spectroscopy (EIS) measurements. Derived from CVs measurements under different scan rates (Figure S13, Supporting Information), ECSAs of Bi@Sn NPs and Sn NPs were calculated via measuring double layer capacitance (*C*_dl_). Figure S14 in the Supporting Information shows the linear relationship between the differences of charging current density and scan rates for Bi@Sn NPs and Sn NPs. The values of *C*_dl_ were fitted to be 2.6 and 2.3 mF cm^−2^ for Bi@Sn NPs and Sn NPs, respectively. We normalized the *j*_HCOOH_ of Bi@Sn NPs and Sn NPs based on the value of *C*_dl_. The normalized *j*_HCOOH_ of Bi@Sn NPs was larger than that of Sn NPs at all applied potentials (Figure S15, Supporting Information). As such, the difference in activity between Bi@Sn NPs and Sn NPs was independent of ECSA. Meanwhile, Bi@Sn NPs also exhibited higher mass activities than Sn NPs, following the same trend of specific activity (Figure S16, Supporting Information). Figure S17 in the Supporting Information shows the Nyquist plots of Bi@Sn NPs and Sn NPs. The charge transfer resistance (*R*_ct_) (205.8 Ω) of Bi@Sn NPs was smaller than that (326.8 Ω) of Sn NPs. Accordingly, Bi@Sn NPs exhibited a faster Faradaic process than Sn NPs toward CO_2_ electroreduction.

To gain insight into the intrinsic reason for the high catalytic performance of Bi@Sn NPs, we conducted density functional theory (DFT) calculation. Based on the modeling study, we found that there existed 8.5% compressive strain in Sn shell for Bi@Sn NPs owing to the lattice mismatch between Bi core and Sn shell (Figure S18, Supporting Information). Taking the strain effect into consideration, we investigated the Gibbs free energy of CO_2_ reduction and the competing HER process on the Sn slab with 8.5% compressive strain (compressive Sn slab) and pristine Sn slab (Table S2, Supporting Information). Generally, *HCOO and *COOH were considered to be the intermediates for the formation of HCOOH and CO, respectively.^[^ ^34^ ^]^ **Figure** **3**a shows Gibbs free energy of the HCOOH pathway. The Gibbs free energy change (Δ*G*) of the conversion from *HCOO to HCOOH was higher than that of the formation of *HCOO on both compressive Sn slab and pristine Sn slab. Therefore, the conversion from *HCOO to HCOOH served as potential‐limiting step on both compressive Sn slab and pristine Sn slab. Notably, the reaction barrier on compressive Sn slab was 0.54 eV, lower than that (0.75 eV) on pristine Sn slab. Accordingly, Sn with compressive strain facilitated the formation of HCOOH during CO_2_ electroreduction process. We also calculated the Gibbs free energy for each steps involved in the CO_2_ reduction into CO on both compressive Sn slab and pristine Sn slab. As shown in Figure 3b, the formation of *COOH served as potential‐limiting step on both compressive Sn slab and pristine Sn slab. The Δ*G* for the formation of *COOH on compressive Sn slab and pristine Sn slab were 1.35 and 1.19 eV, respectively. This result indicated that the introduction of compressive strain into Sn depressed the formation of CO. Furthermore, the Δ*G* for the formation of *H on compressive Sn slab was 0.58 eV, which was higher than that (0.36 eV) on pristine Sn slab, indicating that the competing HER was suppressed by introducing the compressive strain into Sn (Figure 3c).

**Figure 3:**
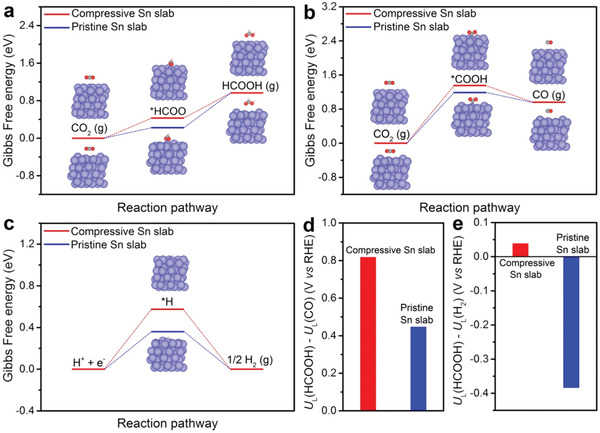
Gibbs free energy diagrams for a) CO_2_ reduction to HCOOH, b) CO_2_ reduction to CO, and c) H_2_ evolution on both compressive Sn slab and pristine Sn slab. The blue, red, black, and pink spheres represent Sn, O, C, and H atoms, respectively. d) The difference in limiting potentials between CO_2_ reduction into HCOOH and CO_2_ reduction into CO on both compressive Sn slab and pristine Sn slab. e) The difference in limiting potentials between CO_2_ reduction into HCOOH and H_2_ evolution on both compressive Sn slab and pristine Sn slab.

To investigate the origin of the strain‐induced optimization of adsorption energies for reaction intermediates, we calculated the projected density of states (PDOS) of compressive Sn slab and pristine Sn slab (Figure S19, Supporting Information). The p‐band center (with regard to the Fermi level) of compressive Sn surface is −1.57 eV, which was 0.1 eV lower than that (−1.47 eV) of pure Sn surface. The downward shift of p‐band center decreases the antibonding states above the Fermi level, resulting in weaker adsorption bonding. Accordingly, the weakened adsorption of intermediates was further confirmed by the shortened bond length between adsorbed species and the reaction sites on compressive Sn surface (Table S3, Supporting Information). In addition, based on the analysis of DFT results, the conversion from *HCOO to HCOOH serves as the potential‐limiting step on both compressive Sn slab and pristine Sn slab. As such, the weakened adsorption strength of *HCOO contributes to the decreased energy barrier for the formation of HCOOH, thus promoting the catalytic performance. Furthermore, the conversion from CO_2_ to *COOH and the formation of *H serve as the potential‐limiting step on both compressive Sn slab and pristine Sn slab. The weakened bonding strength of reactions intermediates (*COOH, *H) results in the suppression of CO and H_2_ production.

The thermodynamic limiting potentials between target product and byproduct is an important way to understand the competing mechanism.^[^ ^35^, ^36^ ^]^ The thermodynamic limiting potentials were donated as *U*_L_(target product) − *U*_L_(byproduct), where *U*_L_ = −Δ*G*/*e*, and the Δ*G* is the value of Gibbs free energy change for the potential‐limiting step. A more positive value of *U*_L_(target product) − *U*_L_(byproduct) corresponds to higher selectivity for target product. As shown in Figure 3d, the value of *U*_L_(HCOOH) − *U*_L_(CO) for compressive Sn slab was 0.82 V, which was higher than that (0.45 V) for pristine Sn slab. The theoretical analysis was in good agreement with the experimental results of the enhanced selectivity for HCOOH over Bi@Sn NPs relative to that over Sn NPs. In addition, compressive Sn slab also exhibited a more positive *U*_L_(HCOOH) − *U*_L_(H_2_) value (0.04 V) than pristine Sn slab (−0.38 V) (Figure 3e). As such, the more positive values of *U*_L_(HCOOH) − *U*_L_(CO) and *U*_L_(HCOOH) − *U*_L_(H_2_) for compressive Sn slab relative to pristine Sn slab, contributed to the enhanced selectivity for CO_2_ electroreduction into HCOOH over Bi@Sn NPs.

Considering that HCOOH is a profitable production with great economic benefits, it is suggested that the economically compelling application of HCOOH required the minimum *j* of −200.0 mA cm^−2^ with FE_HCOOH_ of 90%, and energy efficiencies exceeding 50% in a wide potential range.^[^ ^15^ ^]^ Such a large *j* was difficult to achieve using the traditional H‐cell system due to the mass‐transfer limitation of CO_2_ in aqueous electrolyte. To this end, we conducted CO_2_ electroreduction over Bi@Sn NPs on gas diffusion electrode (GDE) using a flow‐cell system in 2.0 m KHCO_3_ (**Figure** **4**a; Figure S20, Supporting Information). Chronopotentiometric measurements were conducted to evaluate the catalytic performance of Bi@Sn NPs. The FE_HCOOH_ exceeded 92% for Bi@Sn NPs at all applied *j* from −25.0 to −250.0 mA cm^−2^ with corresponding potential ranging from −0.81 to −1.15 V versus RHE (Figure 4b,c). Meanwhile, the energy efficiency was higher than 55% for Bi@Sn NPs when *j* ranged from −25.0 to −250.0 mA cm^−2^ (Figure 4d). Besides, Bi@Sn NPs showed 1% increase for potential and 2% decay for FE_HCOOH_ during the 8 h durability test at *j* of −200.0 mA cm^−2^ (Figure 4e). We further compared the FE_HCOOH_ and *j*_HCOOH_ of Bi@Sn NPs with other reported Sn‐based catalysts (Table S4, Supporting Information). Possessing the high *j*_HCOOH_ and FE_HCOOH_ at low applied potential, Bi@Sn NPs represented one of the best Sn‐based catalysts for the electroreduction CO_2_ into HCOOH up to now.

**Figure 4:**
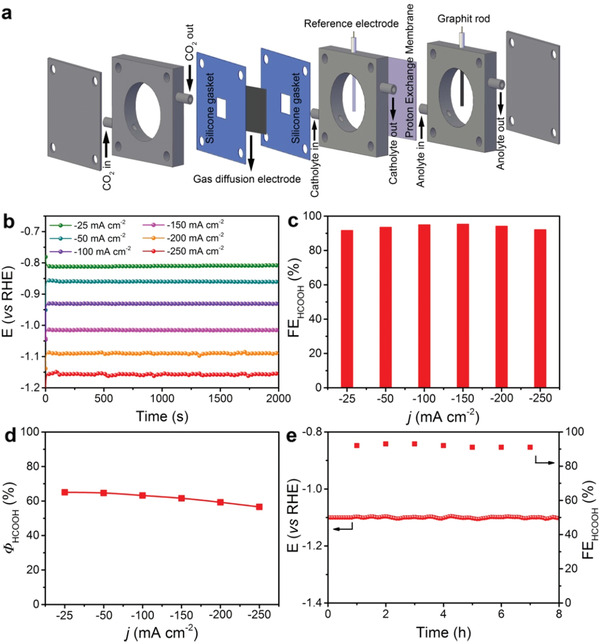
a) Schematic illustration of the flow‐cell system. b) Plots of potential–time curves with *iR*‐correction, c) FE_HCOOH_, and d) *Φ*_HCOOH_ of Bi@Sn NPs at the *j* ranging from −25.0 to −250.0 mA cm^−2^ in 2.0 m KHCO_3_. e) Durability tests of Bi@Sn NPs in the flow‐cell system at *j* of −200.0 mA cm^−2^ for 8 h.

In conclusion, we developed Bi@Sn NPs with core–shell structure as efficient catalyst for CO_2_ electroreduction into HCOOH. Bi@Sn NPs achieved an FE_HCOOH_ of 92% with *j* as high as −250.0 mA cm^−2^ using flow‐cell system with 2.0 m KHCO_3_ as electrolyte. Theoretical studies indicate that energy barrier of the potential‐limiting step for the formation of HCOOH was decreased owing to the compressive strain in Sn shell, resulting in the enhanced catalytic performance. This work not only developed promising catalysts toward CO_2_ electroreduction into HCOOH, but also provided a strategy for the rational design of highly efficient electrocatalysts by regulating the lattice strain.

## Conflict of Interest

The authors declare no conflict of interest.

## Supporting information

Supporting Information

## References

[1] M. G. Kibria , J. P. Edwards , C. M. Gabardo , C. T. Dinh , A. Seifitokaldani , D. Sinton , E. H. Sargent , Adv. Mater. 2019, 31, 1807166. 10.1002/adma.201807166 31095806

[2] L. Zhang , Z.‐J. Zhao , J. Gong , Angew. Chem., Int. Ed. 2017, 56, 11326. 10.1002/anie.201612214 28168799

[3] Z.‐L. Wang , C. Li , Y. Yamauchi , Nano Today 2016, 11, 373.

[4] C. Kim , T. Möller , J. Schmidt , A. Thomas , P. Strasser , ACS Catal. 2019, 9, 1482.

[5] J. Gu , F. Héroguel , J. Luterbacher , X. Hu , Angew. Chem., Int. Ed. 2018, 57, 2943. 10.1002/anie.201713003 29356272

[6] Y. Fang , J. C. Flake , J. Am. Chem. Soc. 2017, 139, 3399. 28182409 10.1021/jacs.6b11023

[7] Y. Zheng , A. Vasileff , X. Zhou , Y. Jiao , M. Jaroniec , S.‐Z. Qiao , J. Am. Chem. Soc. 2019, 141, 7646. 30986349 10.1021/jacs.9b02124

[8] Q. H. Low , N. W. X. Loo , F. Calle‐Vallejo , B. S. Yeo , Angew. Chem., Int. Ed. 2019, 58, 2256. 10.1002/anie.201810991 30565358

[9] J. Gu , C.‐S. Hsu , L. Bai , H. M. Chen , X. Hu , Science 2019, 364, 1091. 31197014 10.1126/science.aaw7515

[10] K. Jiang , R. B. Sandberg , A. J. Akey , X. Liu , D. C. Bell , J. K. Nørskov , K. Chan , H. Wang , Nat. Catal. 2018, 1, 111.

[11] Z. Geng , X. Kong , W. Chen , H. Su , Y. Liu , F. Cai , G. Wang , J. Zeng , Angew. Chem., Int. Ed. 2018, 57, 6054. 10.1002/anie.201711255 29645366

[12] X. Zheng , P. D. Luna , F. P. García de Arquer , B. Zhang , N. Becknell , M. B. Ross , Y. Li , M. N. Banis , Y. Li , M. Liu , O. Voznyy , C. T. Dinh , T. Zhuang , P. Stadler , Y. Cui , X. Du , P. Yang , E. H. Sargent , Joule 2017, 1, 794.

[13] T. Reda , C. M. Plugge , N. J. Abram , J. Hirst , Proc. Natl. Acad. Sci. USA 2008, 105, 10654. 18667702 10.1073/pnas.0801290105 PMC2491486

[14] D. Mellmann , P. Sponholz , H. Junge , M. Beller , Chem. Soc. Rev. 2016, 45, 3954. 27119123 10.1039/c5cs00618j

[15] S. Verma , B. Kim , H. R. M. Jhong , S. Ma , P. J. A. Kenis , ChemSusChem 2016, 9, 1972. 27345560 10.1002/cssc.201600394

[16] A. Klinkova , P. D. Luna , C.‐T. Dinh , O. Voznyy , E. M. Larin , E. Kumacheva , E. H. Sargent , ACS Catal. 2016, 6, 8115.

[17] Q. Zhu , J. Ma , X. Kang , X. Sun , H. Liu , J. Hu , Z. Liu , B. Han , Angew. Chem., Int. Ed. 2016, 55, 9012. 10.1002/anie.201601974 27311592

[18] C. H. Lee , M. W. Kanan , ACS Catal. 2015, 5, 465.

[19] Z. Chen , N. Wang , S. Yao , L. Liu , J. CO2 Util. 2017, 22, 191.

[20] Y. Hori , Mod. Aspects Electrochem. 2008, 42, 89.

[21] J. Zhang , R. Yin , Q. Shao , T. Zhu , X. Huang , Angew. Chem., Int. Ed. 2019, 58, 5609. 10.1002/anie.201900167 30815992

[22] Y. Zhao , J. Liang , C. Wang , J. Ma , G. G. Wallace , Adv. Energy Mater. 2018, 8, 1702524.

[23] X. Zheng , Y. Ji , J. Tang , J. Wang , B. Liu , H.‐G. Steinrück , K. Lim , Y. Li , M. F. Toney , K. Chan , Y. Cui , Nat. Catal. 2019, 2, 55.

[24] R. Daiyan , E. C. Lovell , N. M. Bedford , W. H. Saputera , K.‐H. Wu , S. Lim , J. Horlyck , Y. H. Ng , X. Lu , R. Amal , Adv. Sci. 2019, 6, 1900678. 10.1002/advs.201900678 PMC6755522 31559127

[25] Q. Li , J. Fu , W. Zhu , Z. Chen , B. Shen , L. Wu , Z. Xi , T. Wang , G. Lu , J.‐J. Zhu , S. Sun , J. Am. Chem. Soc. 2017, 139, 4290. 28291338 10.1021/jacs.7b00261

[26] A. Zhang , R. He , H. Li , Y. Chen , T. Kong , K. Li , H. Ju , J. Zhu , W. Zhu , J. Zeng , Angew. Chem., Int. Ed. 2018, 57, 10954. 10.1002/anie.201806043 29953722

[27] S. Liu , J. Xiao , X. F. Lu , J. Wang , X. Wang , X. W. Lou , Angew. Chem., Int. Ed. 2019, 58, 8499. 10.1002/anie.201903613 30974035

[28] F. Lei , W. Liu , Y. Sun , J. Xu , K. Liu , L. Liang , T. Yao , B. Pan , S. Wei , Y. Xie , Nat. Commun. 2016, 7, 12697. 27585984 10.1038/ncomms12697 PMC5025773

[29] R. Daiyan , X. Lu , W. H. Saputera , Y. H. Ng , R. Amal , ACS Sustainable Chem. Eng. 2018, 6, 1670.

[30] W. Zhang , Y. Hu , L. Ma , G. Zhu , P. Zhao , X. Xue , R. Chen , S. Yang , J. Ma , J. Liu , Z. Jin , Nano Energy 2018, 53, 808.

[31] W. Deng , L. Zhang , L. Li , S. Chen , C. Hu , Z.‐J. Zhao , T. Wang , J. Gong , J. Am. Chem. Soc. 2019, 141, 2911. 30715865 10.1021/jacs.8b13786

[32] G. Samjeské , A. Miki , S. Ye , M. Osawa , J. Phys. Chem. B 2006, 110, 16559. 16913790 10.1021/jp061891l

[33] B. Kumar , V. Atla , J. P. Brian , S. Kumari , T. Q. Nguyen , M. Sunkara , J. M. Spurgeon , Angew. Chem., Int. Ed. 2017, 56, 3645. 10.1002/anie.201612194 28229519

[34] W. Ma , S. Xie , X.‐G. Zhang , F. Sun , J. Kang , Z. Jiang , Q. Zhang , D.‐Y. Wu , Y. Wang , Nat. Commun. 2019, 10, 892. 30792388 10.1038/s41467-019-08805-x PMC6385284

[35] W. Ren , X. Tan , W. Yang , C. Jia , S. Xu , K. Wang , S. C. Smith , C. Zhao , Angew. Chem., Int. Ed. 2019, 58, 6972. 10.1002/anie.201901575 30920151

[36] X. Li , W. Bi , M. Chen , Y. Sun , H. Ju , W. Yan , J. Zhu , X. Wu , W. Chu , C. Wu , C. Wu , Y. Xie , J. Am. Chem. Soc. 2017, 139, 14889. 28992701 10.1021/jacs.7b09074

