# Supplementary material for: Bi@Sn Core–Shell Structure with Compressive Strain Boosts the Electroreduction of CO2 into Formic Acid
Source: Adv Sci (Weinh). 2020 Oct 1;7(22):1902989. doi: 10.1002/advs.201902989 (PMC7675058; doi:10.1002/advs.201902989)
Supplement: Supplementary file 1 — Supporting Information [file ADVS-7-1902989-s001.pdf]

WILEY-VCH

© 2020 Wiley-VCH GmbH

ADVANCED SCIENCE
Open Access

Supporting Information

for *Adv. Sci.*, DOI: 10.1002/advs.201902989

Bi@Sn Core-Shell Structure with Compressive Strain Boosts the Electroreduction of CO_2 into Formic Acid

Yulin Xing, Xiangdong Kong, Xu Guo, Yan Liu, Qiuyao Li, Yuzhe Zhang, Yelin Sheng, Xupeng Yang, Zhigang Geng, and Jie Zeng**

Supplementary Information for

Bi@Sn Core-Shell Structure with Compressive Strain Boosts the Electroreduction of CO_2 into Formic Acid

Yulin Xing, Xiangdong Kong, Xu Guo, Yan Liu, Qiuyao Li, Yuzhe Zhang, Yelin Sheng, Xupeng Yang, Zhigang Geng,* and Jie Zeng*

Hefei National Laboratory for Physical Sciences at the Microscale, CAS Key Laboratory of Strongly-Coupled Quantum Matter Physics, Key Laboratory of Surface and Interface Chemistry and Energy Catalysis of Anhui Higher Education Institutes, Department of Chemical Physics, University of Science and Technology of China, Hefei, Anhui 230026, P. R. China

*Corresponding author. E-mail: gengzg@ustc.edu.cn (Z.G.); E-mail: zengj@ustc.edu.cn (J.Z.).

Experimental section

Chemicals and materials. Sodium stannate tetrahydrate ($Na_2SnO_3 \cdot 4H_2O$, 98.0%), bismuth nitrate pentahydrate ($Bi(NO_3)_3 \cdot 5H_2O$, 99.0%), tin chloride dihydrate ($SnCl_2 \cdot 2H_2O$, 98.0%), sodium hydroxide (NaOH, 96.0%), sodium borohydride ($NaBH_4$, 98.0%), sodium formate dihydrate ($HCOONa \cdot 2H_2O$, 99.5%), and ethanol (C_2H_5OH, 99.7%) were all purchased from Sinopharm Chemical Reagent Co. Ltd. (Shanghai, China). 1-Propanesulfonic acid 3-(trimethylsilyl) sodium salt (DSS), Nafion solution (5 wt%), and Nafion 115 film were purchased from Sigma-Aldrich. The deionized (DI) water was produced using a Millipore Milli-Q grade with a resistivity of 18.2 MΩ cm. All the chemicals were used as received without any further purification.

Synthesis of $Bi_2Sn_2O_7$ NPs. In a typical synthesis, 1.139 g of $Na_2SnO_4 \cdot 4H_2O$ was dissolved in 36 mL of deionized water. Then, 1.940 g of $Bi(NO_3)_3 \cdot 5H_2O$ was added into the above solution under vigorous stirring. The pH value of the solution was adjusted to 12 using NaOH solution (5 M). After stirring for 10 minutes, the solution was transferred into a 45-mL Teflon-lined stainless steel autoclave at 180 ^oC for 24 h. After the autoclave was cooled down naturally to room temperature, the precipitate was washed by centrifuge with ethanol and DI water for each three times. $Bi_2Sn_2O_7$ NPs were obtained after being dried under vacuum at 80 ^oC for overnight.

Synthesis of Sn NPs. The Sn NPs were prepared by reducing $SnCl_2$ with $NaBH_4$ solution. To prepare Sn precursor solution, 38 mg of $SnCl_2 \cdot 2H_2O$ was dissolved in 5 mL of ethanol. Then Sn precursor was added droplet into 10 mL of $NaBH_4$ solution (0.2 M) under stirring. After 30 minutes, the precipitate was washed by centrifuge with ethanol and DI water for each three times. Sn NPs were obtained after being dried under vacuum at 80 ^oC for overnight.

Preparation of Bi@Sn NPs and Sn NPs as working electrode. Typically, 5 mg of $Bi_2Sn_2O_7$ NPs, 5 mg of carbon black, and 20 μL of Nafion solution were dispersed in 1 mL of ethanol by sonicating for 1 h to form a homogeneous ink. Then, 50 μL of the mixture was uniformly spread on carbon paper with an area of 1 × 0.5 cm^2 and was naturally dried. Electrochemical experiments were conducted in an H-cell with a Nafion 115 membrane as the separator. Each compartment of the H-cell contained 30 mL of 0.5 M $KHCO_3$ electrolyte. The graphite rod and

Ag/AgCl electrodes were used as the counter electrode and reference electrode, respectively. Bi@Sn NPs were synthesized via electroreduction of $Bi_2Sn_2O_7$ NPs in 0.5 M $KHCO_3$ at -0.8 V *vs* RHE for 1 h. The prepared carbon-paper electrode directly used as working electrode without further treatment. For Sn NPs, the working electrode was prepared with the same condition except the process of electroreduction.

Electrochemical measurements. The potentials were controlled via an Autolab potentiostat/galvanostat (CHI660E). All potentials were measured against the Ag/AgCl reference electrode and converted to the RHE reference scale on account of the equation: E (*vs* RHE) = E (*vs* Ag/AgCl) + 0.21 V + 0.0591 × pH. The LSV curves were performed in CO_2-saturated and Ar-saturated 0.5 M $KHCO_3$ solution with the scan rate of 10 mV s^{-1} in H-cell system. The cyclic voltammetry (CV) measurement was conducted in CO_2-saturated 0.5 M $KHCO_3$ solution using three-electrode cell equipped with a Ag/AgCl reference electrode, a graphite rod counter electrode and a glassy carbon working electrode (5 mm in diameter). C_{dl} was determined by measuring the capacitive current associated with double-layer charging from the scan-rate dependence of CV. The CV ranged from 0.4 V to 0.5 V *vs* RHE. The C_{dl} was estimated by plotting the Δj ($\Delta j = j_a - j_c$) at 0.45 V *vs* RHE against the scan rates. The slope is twice that of the C_{dl} value. Electrochemical impedance spectroscopy of Bi@Sn NPs and Sn NPs were recorded at -0.5 V *vs* RHE over a frequency range from 1000 kHz to 1 Hz with a sinusoidal voltage amplitude of 5 mV. CO_2 electroreduction was conducted in CO_2-saturated 0.5 M $KHCO_3$ solution in the H-cell system under ambient condition at room temperature. After the feeding gas of CO_2 was purged into 0.5 M $KHCO_3$ solution for at least 30 min to remove residual air in the reservoir, controlled potential electrolysis was performed at each potential for 40 min. The gaseous products of CO_2 electroreduction were monitored via an online gas chromatography (GC) (GC2014, Shimadzu, Japan) equipped with a thermal conductivity detector (TCD) and Molsieve 5A column once every five minutes. The $KHCO_3$ solution after electrolysis was collected and analyzed on a 400 MHz NMR spectrometer to quantify liquid products. Standard curve was made by using $HCOONa \cdot 2H_2O$ and the internal standard (DSS). 0.5 mL of the $KHCO_3$ solution after electrolysis was mixed with the addition of 0.1 mL of D_2O and 0.1 mL of 6 mM DSS solution as an internal standard. The peak-area ratio of the HCOOH peak to the DSS peak was compared to the standard curve to quantify the concentration of HCOOH.

Electrolysis in the flow-cell system. The cathode and anode chambers were each 15 cm^3 in volume. Each chamber had an inlet and an outlet for electrolyte. Ag/AgCl reference electrode was located inside the cathode chamber. The windows for electrolysis were set to 1 cm × 1 cm. The prepared carbon-paper electrode in H-cell system was directly used as GDE. Silicone gasket with a 1 cm × 1 cm window was placed between GDE and cathode chamber for sealing. CO_2 electroreduction was conducted in 2.0 M $KHCO_3$ solution in the flow-cell system under ambient condition at room temperature. During the measurements, CO_2 gas was directly fed to the cathode GDE at a rate of 20 sccm. The electrolyte was forced to continuously circulate through the chamber at a rate of 10 sccm.

The calculation method for the Faradaic efficiency.

The Faradaic efficiency for HCOOH production was calculated at a given potential as follows:

$$\mathrm{FE_{HCOOH}} = C_{\mathrm{HCOOH}} \times V \times \mathrm{N} \times \mathrm{F} / Q$$

C_{HCOOH}, the concentration of HCOOH.

V, the volume of the electrolyte.

N, the number of electron transferred for product formation, which is 2 for HCOOH.

F, Faraday constant, 96485 C mol^{-1}.

Q, quantity of electric charge integrated by *i*-*t* curve.

The calculation method for the yield rate of HCOOH.

The yield rate of HCOOH was calculated at a given potential as follow:

$$v_{\mathrm{HCOOH}} = (C_{\mathrm{HCOOH}} \times V) / (\mathrm{S} \times \mathrm{t})$$

v_{HCOOH}, the yield rate of HCOOH.

C_{HCOOH}, the concentration of HCOOH.

V, the volume of the electrolyte.

S, the geometric area of the electrode.

t, the reaction time.

The calculation method for the energy efficiency for the conversion of CO_2 into HCOOH.

The energy efficiency for the conversion of CO_2 into HCOOH was calculated at a given potential as follow:

$$\Phi_{HCOOH} = (FE \times \Delta E^0_{HCOOH}) / \Delta E_{HCOOH}$$

Φ_{HCOOH}, the energy efficiency for the conversion of CO_2 into HCOOH.
FE, the Faradaic efficiency for HCOOH.
ΔE^0_{HCOOH}, the difference between the standard half reaction potentials for water oxidation (1.23 V *vs* RHE) and the reduction of CO_2 into HCOOH (-0.2 V *vs* RHE).
ΔE_{HCOOH}, the difference between the standard water oxidation potential and the working potential at the cathode.

Density functional theory (DFT) calculation. As evidenced by the XRD pattern, Bi@Sn NPs exhibited a larger $I_{Bi(012)}/I_{Bi(104)}$ value (4.18) than that (2.95) of standard rhombohedral Bi. Therefore, the Bi core in Bi@Sn NPs exhibited preferred orientations of (012) facets. As such, we constructed the structure model of Bi/Sn interface based on the Bi (012) plane with different Sn planes. Specifically, based on the XRD pattern of Bi@Sn NPs, three different planes of Sn including (200), (101), and (211) were taken into consideration. Figure S18 illustrates the lattice constants of Sn (101), Sn (200), Sn (211), and Bi (012) planes, respectively. Owing to the large difference in lattice constants, the Sn (200) and (211) planes could not epitaxially grow on Bi (012) plane. Whereas, Sn (101) plane could epitaxially grow on Bi (012) plane. Specifically, in *x*-direction of the interface, the unit of Sn (9.43 Å) in $[1\bar{1}\bar{1}]$ direction fits well with two units of Bi (9.43 Å) in $[\bar{2}00]$ direction. The lattice mismatch exists along the *y* direction between two units of Sn (8.74 Å) in $[13\bar{1}]$ direction and one unit of Bi (7.99 Å) in $[02\bar{1}]$ direction, resulting in 8.5% compressive strain along the $[13\bar{1}]$ direction in Sn (101) plane. The compressive strain was taken into consideration in the DFT calculations.

DFT calculation was carried out using the Vienna *ab initio* Simulation Package (VASP).[S1] The projector augmented wave method in conjugation with a generalized gradient approximation of exchange-correlation functional in the Perdew-Burke-Ernzerhof (PBE) form was adopted.[S2] The one-electron wave functions were expanded using a plane-wave basis set with an energy cut off of 400 eV.[S3] Sn (101) slabs with (2 × 2) surface unit cells and 4 atomic layers was performed. In the geometric optimization, atomic positions were fully relaxed until the associated forces are less than 0.02 eV/Å. 4 × 5 × 1 Monkhorst-Pack sampled k-points were used in all of slabs system. For projected density of states (PDOS) plotting, a larger set of k-points (8 × 10 × 1) were used. A

dipole correction was imposed to eliminate the error induced by the artificial electrostatic interaction between the surface dipole moments of asymmetric repeated slabs. A vacuum region greater than 14 Å was applied to avoid interactions between the neighboring slabs caused by the periodic boundary conditions.

The reaction steps considered for the electroreduction of CO_2 into HCOOH, CO, and the competing HER are as follows:

HCOOH:

(1) $CO_2 + * + H^+ + e^- \rightarrow *HCOO$

(2) $*HCOO + H^+ + e^- \rightarrow HCOOH(g) + *$

CO:

(3) $CO_2 + * + H^+ + e^- \rightarrow *COOH$

(4) $*COOH + H^+ + e^- \rightarrow CO(g) + H_2O + *$

H_2:

(5) $* + H^+ + e^- \rightarrow *H$

(6) $*H \rightarrow 1/2H_2(g) + *$

The Gibbs free energy for each step involved in the CO_2 reduction and HER at 0 V *vs* RHE on the compressive Sn slab and pristine Sn slab was calculated as

$$G = E_{DFT} + ZPVE - TS_{vib}$$

E is the DFT-optimized total energy, *ZPVE* is the zero point energy, TS_{vib} respect the vibrational entropy corrections, which were calculated through the harmonic-oscillator approximation.

Instrumentations. XRD patterns were recorded by using a Philips X'Pert Pro Super diffractometer with Cu-Kα radiation (λ=1.54178 Å). TEM images were taken using a Hitachi H-7650 transmission electron microscope at an acceleration voltage of 100 kV. HRTEM, HAADF-STEM, and EDX mapping were carried out on a JEOL ARM-200F field-emission transmission electron microscope operating at 200 kV accelerating voltage. The liquid products were quantified by nuclear magnetic resonance (Bruker AVANCE AV III 400) spectroscopy. *In-situ* attenuated total reflection infrared (ATR-IR) spectroscopy was carried out on a Nicolet iS50 with a wavenumber resolution of 8 cm^{-1} at room temperature.

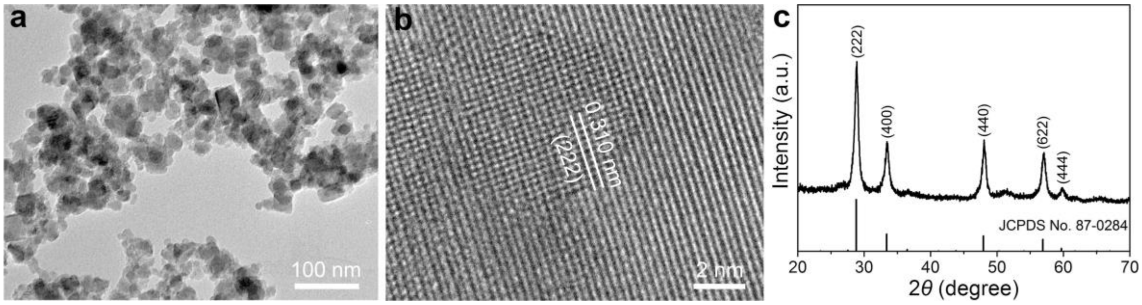

Figure S1. (a) TEM image, (b) HRTEM image, and (c) XRD pattern of $Bi_2Sn_2O_7$ NPs.

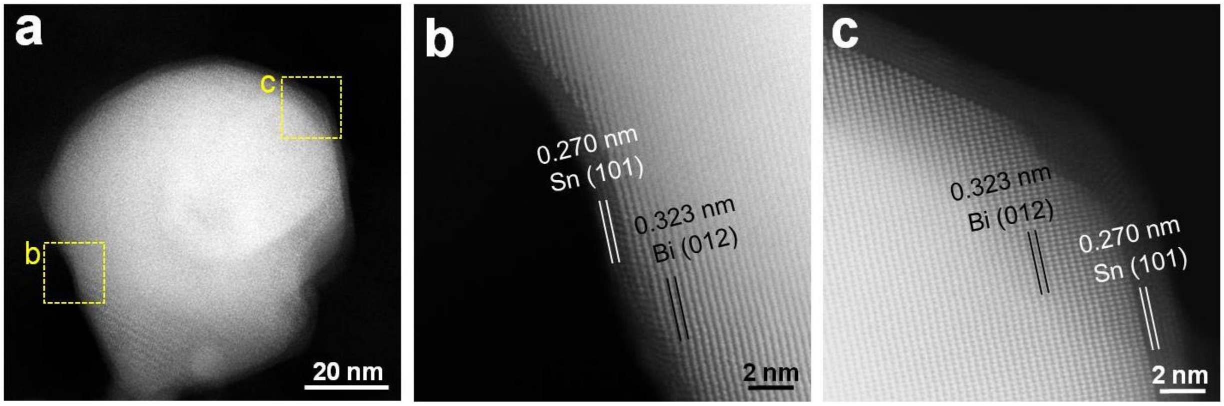

Figure S2. (a) HAADF-STEM image of an individual Bi@Sn NP. (b) and (c) magnified HAADF-STEM images from the selected areas in (a).

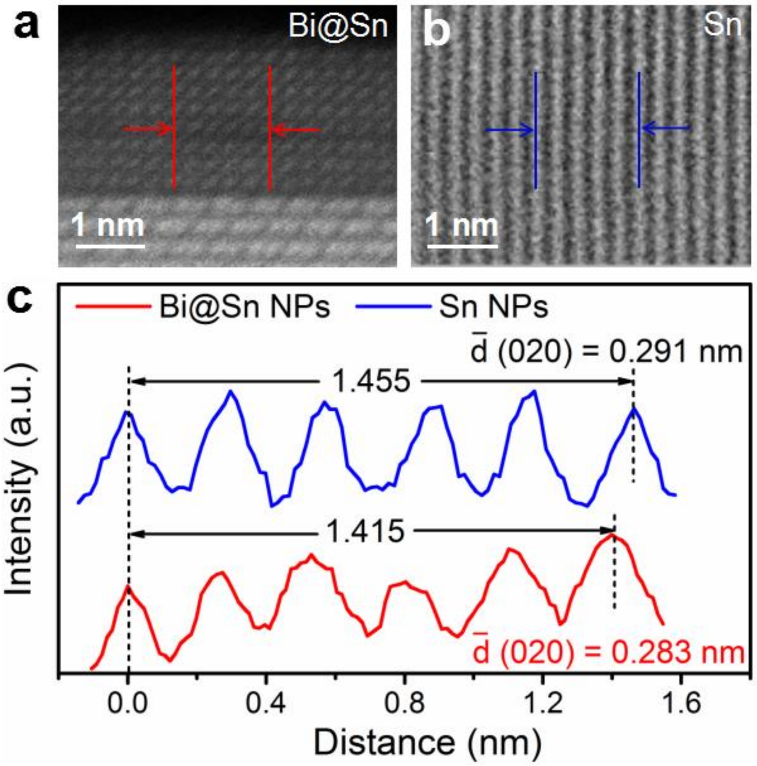

Figure S3. HAADF-STEM images of (a) Bi@Sn NP and (b) Sn NP. The red and blue bars denote the areas analyzed for (020) spacing. (c) The integrated pixel intensities of Sn in Bi@Sn NP and Sn NP along (020) spacing directions.The peaks and valleys represent the atoms and gaps, respectively. The interplanar spacing of Sn (020) plane is averaged over five atomic layers for high accuracy. The interplanar spacing of Sn (020) plane for Sn NP is measured to be 0.291 nm, whereas it is 0.283 nm for Sn shell in Bi@Sn NP.

Table S1. Comparison of diffraction angles in the XRD patterns for Bi@Sn NPs with Sn (JCPDS No. 89-2761) and Bi (JCPDS No. 85-1330).

Samples	2θ (degree) for diffraction peaks					
	Bi (012)	Bi (104)	Bi (202)	Sn (200)	Sn (101)	Sn (211)
Bi@Sn NPs	27.22	38.02	48.80	30.74	32.09	44.98
Sn (JCPDS No.89-2761)	/	/	/	30.63	32.01	44.89
Bi (JCPDS No.85-1330)	27.25	38.09	48.83	/	/	/

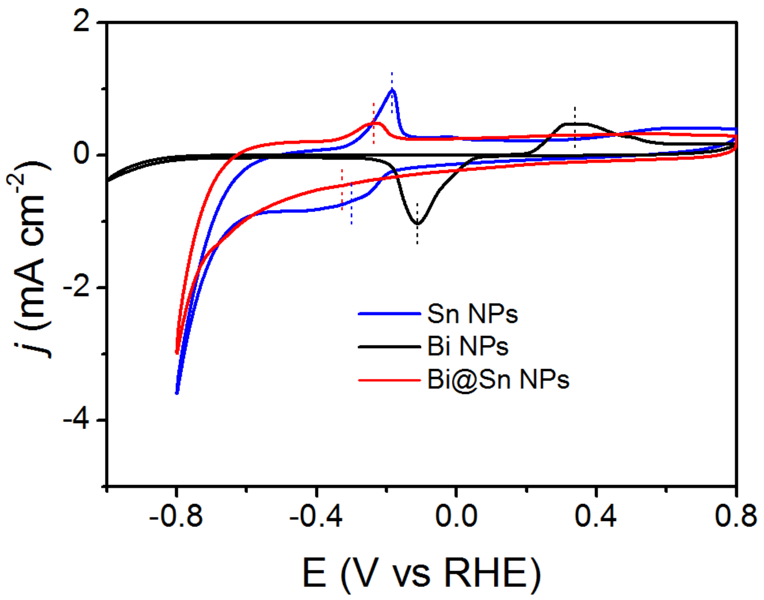

Figure S4. CV curves of Sn NPs, Bi NPs, and Bi@Sn NPs in Ar-saturated 0.5 M $KHCO_3$ electrolyte at scan rate of 20 mV s^{-1}. Bi NPs exhibited an obvious couple of redox peaks at -0.11 V *vs* RHE (Bi_2O_3 to Bi) and 0.34 V *vs* RHE (Bi to Bi_2O_3). In comparison, Bi@Sn NPs showed no redox peaks related to Bi NPs. Meanwhile, Bi@Sn NPs exhibited a similar couple of redox peaks to that of Sn NPs.

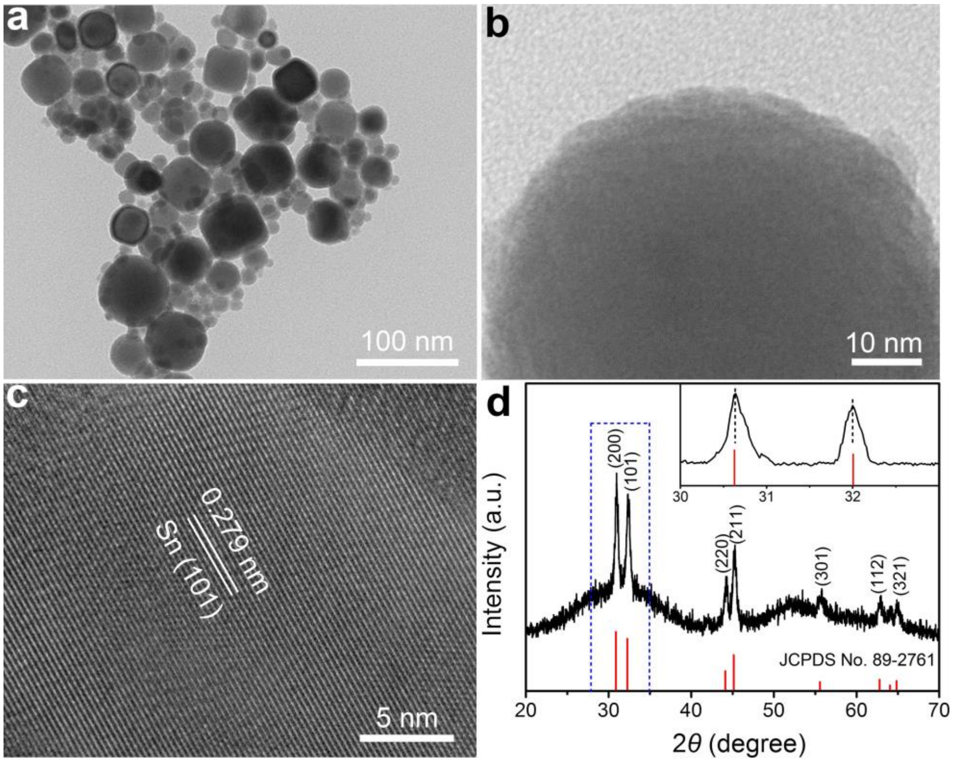

Figure S5. (a) TEM image, (b) magnified TEM image, (c) HRTEM image, and (d) XRD pattern of Sn NPs, the corresponding magnified XRD pattern of Sn NPs is given in the inset.

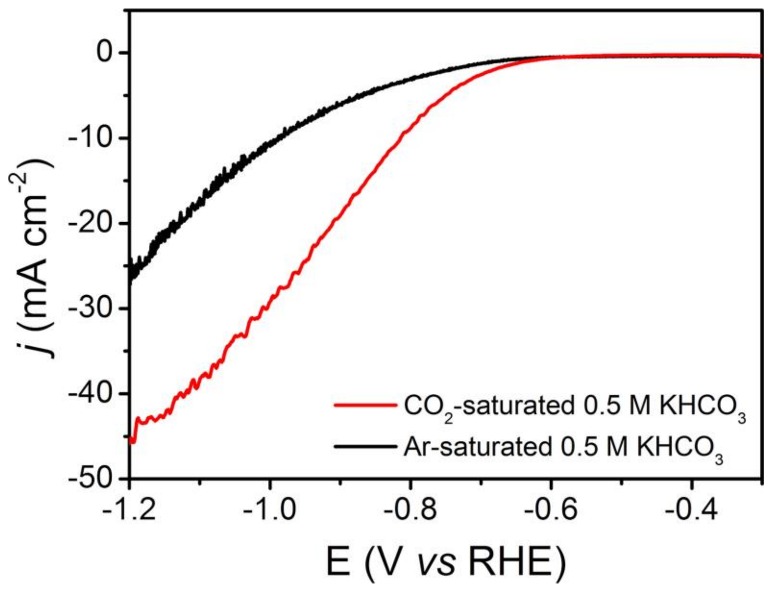

Figure S6. LSV curves of Bi@Sn NPs were performed in CO_2-saturated and Ar-saturated 0.5 M $KHCO_3$.

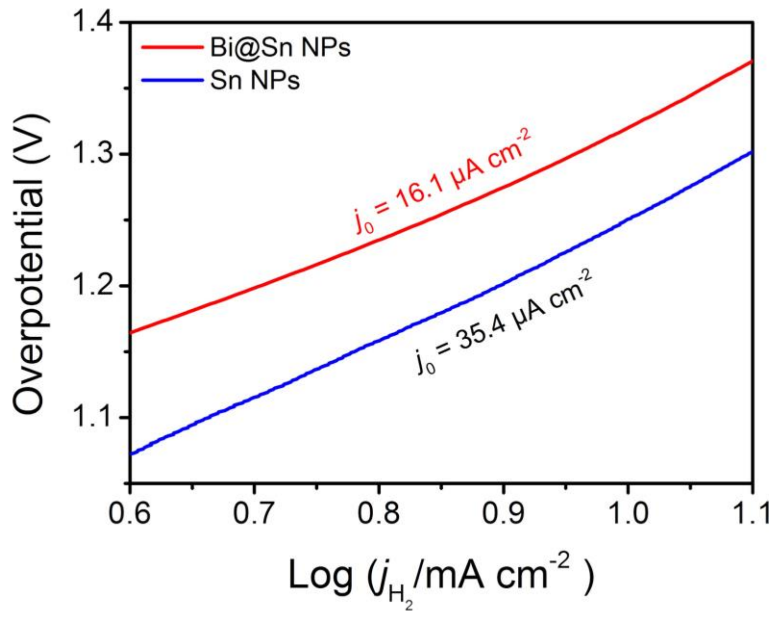

Figure S7. Tafel plots of Bi@Sn NPs and Sn NPs in Ar-saturated 0.5 M $KHCO_3$ electrolyte. The exchange current densities (j_0) for HER were derived from the intercept of the linear region in Tafel plots.

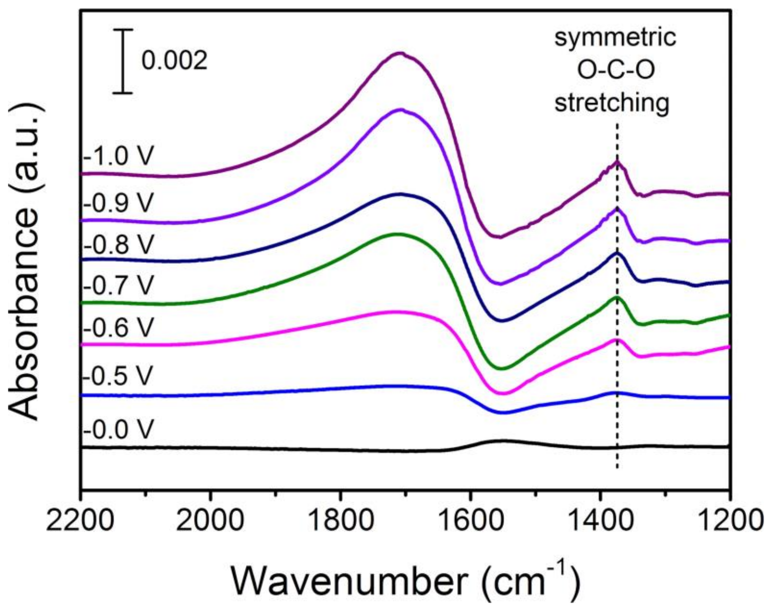

Figure S8. The *in-situ* ATR-IR spectra for Bi@Sn NPs recorded at different potentials in CO_2-saturated 0.5 M $KHCO_3$ electrolyte.

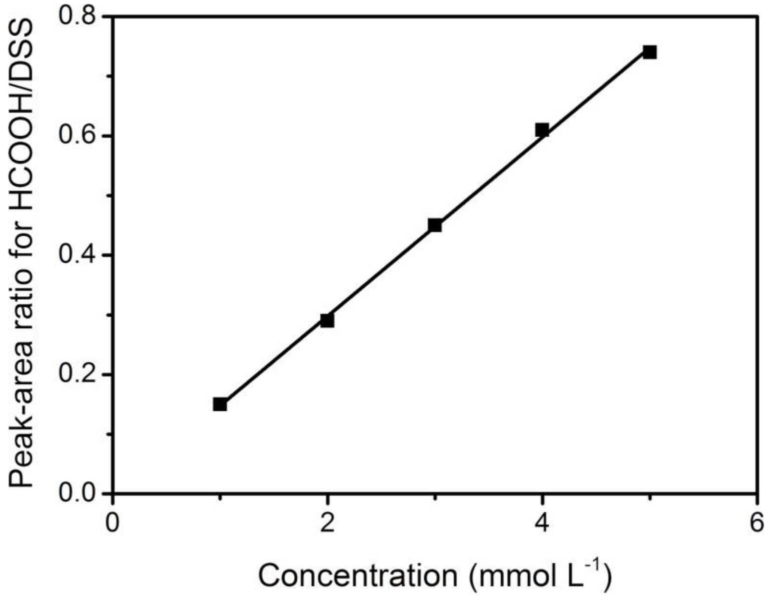

Figure S9. Plot of concentration with peak-area ratio for HCOOH/DSS. The standard curve showed good linear relation of peak-area ratio for HCOOH/DSS with HCOOH concentration ($y = 0.150\ x$, $R^2 = 0.999$).

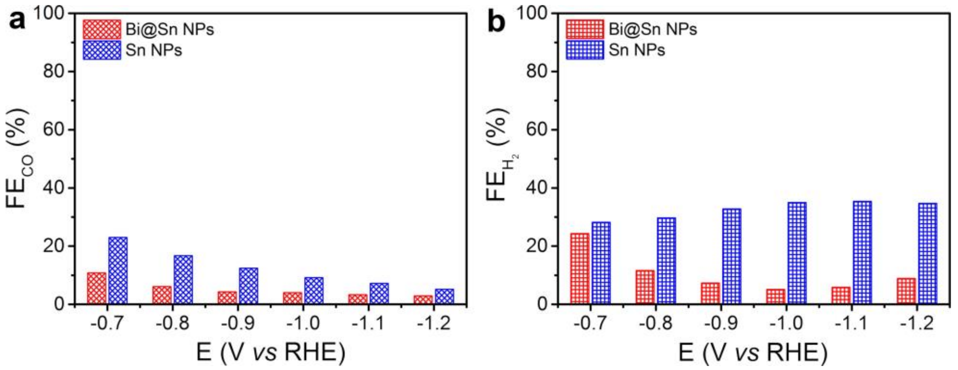

Figure S10. (a) FE_{CO} and (b) FE_{H_2} of Bi@Sn NPs and Sn NPs at different applied potentials.

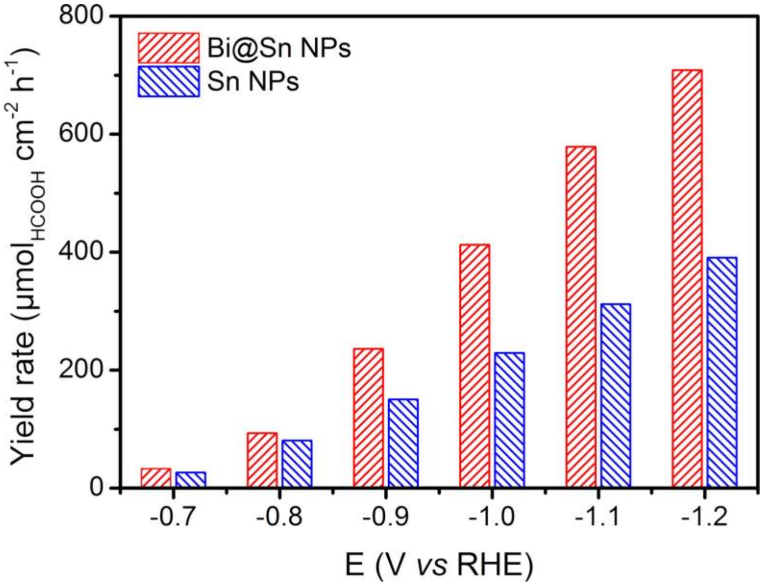

Figure S11. Yield rate for HCOOH of Bi@Sn NPs and Sn NPs at all applied potentials towards CO_2 electroreduction.

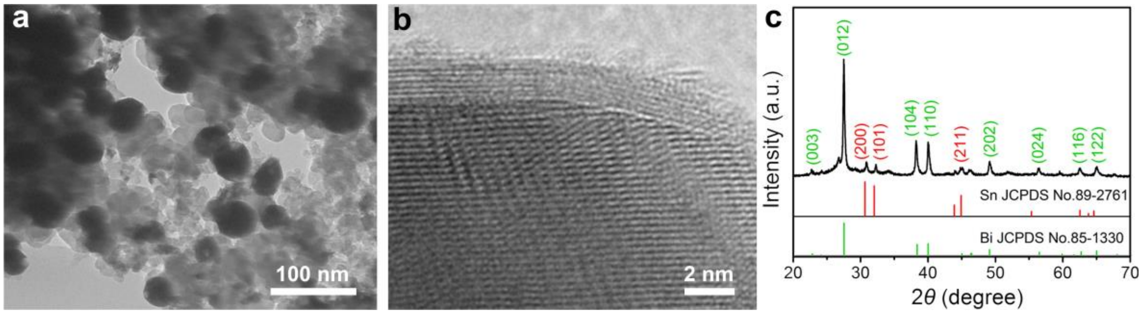

Figure S12. (a) TEM image, (b) HRTEM image, and (c) XRD pattern of Bi@Sn NPs after electrolysis.

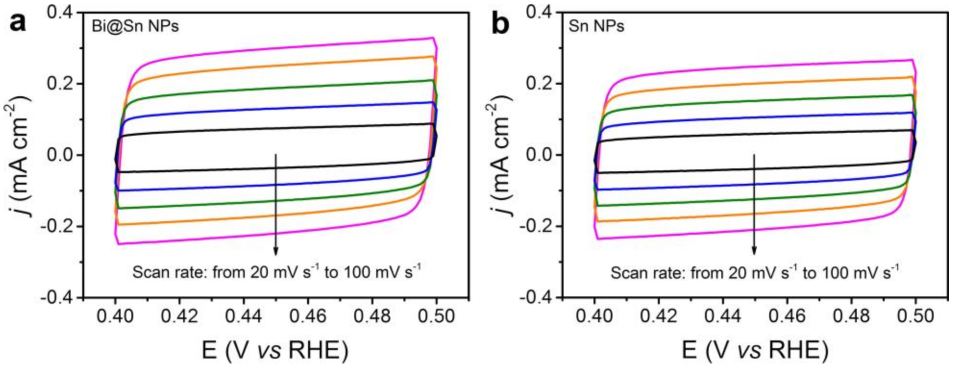

Figure S13. CV curves of (a) Bi@Sn NPs and (b) Sn NPs at different scan rates.

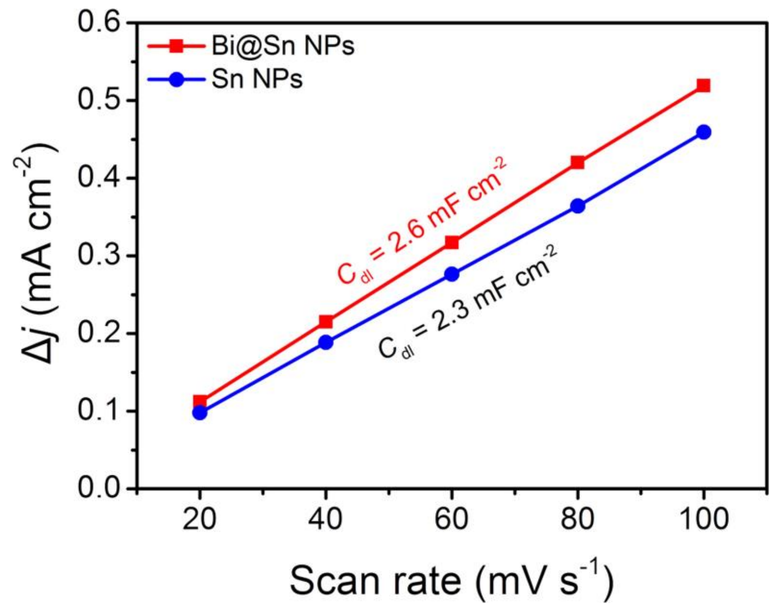

Figure S14. Charging current density differences plotted against scan rates for Bi@Sn NPs and Sn NPs.

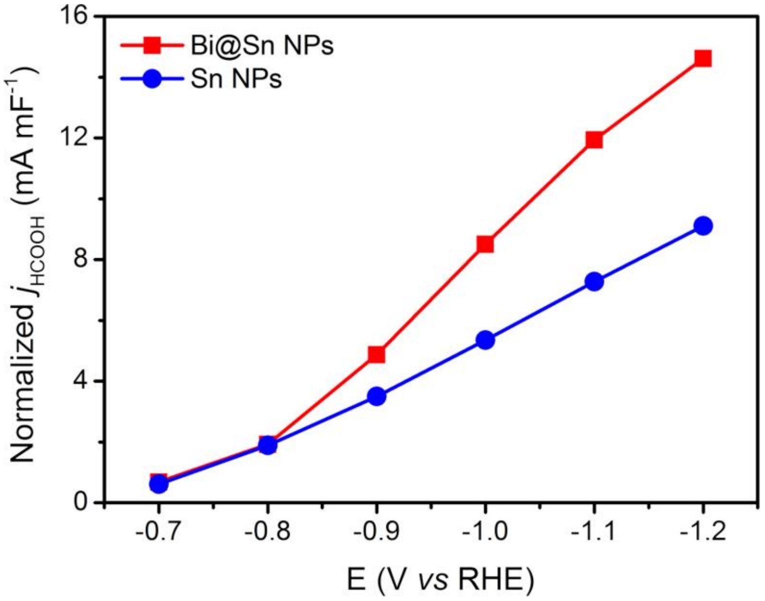

Figure S15. The j_{HCOOH} normalized by the C_{dl} value for Bi@Sn NPs and Sn NPs.

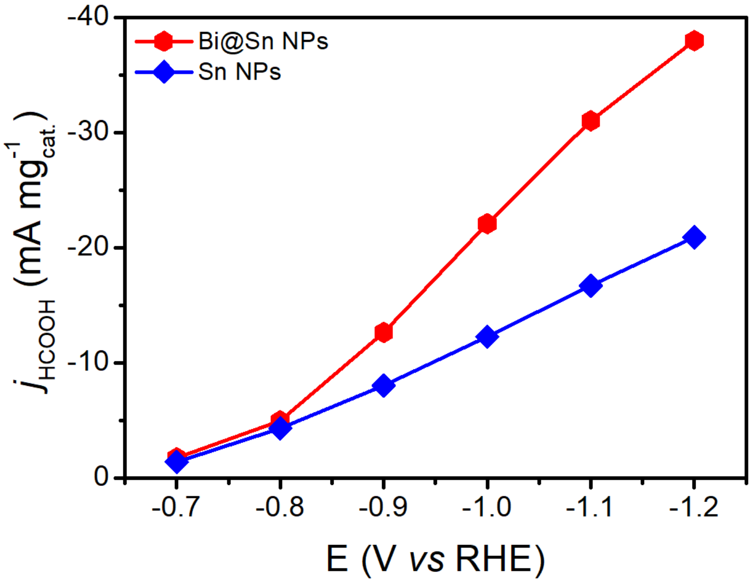

Figure S16. The j_{HCOOH} normalized by the mass of Bi@Sn NPs and Sn NPs at different applied potentials.

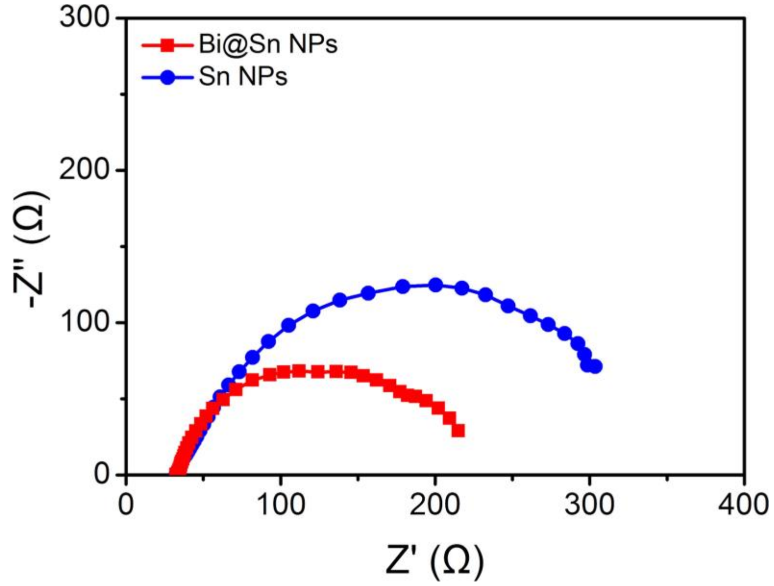

Figure S17. Nyquist plots of Bi@Sn NPs and Sn NPs.

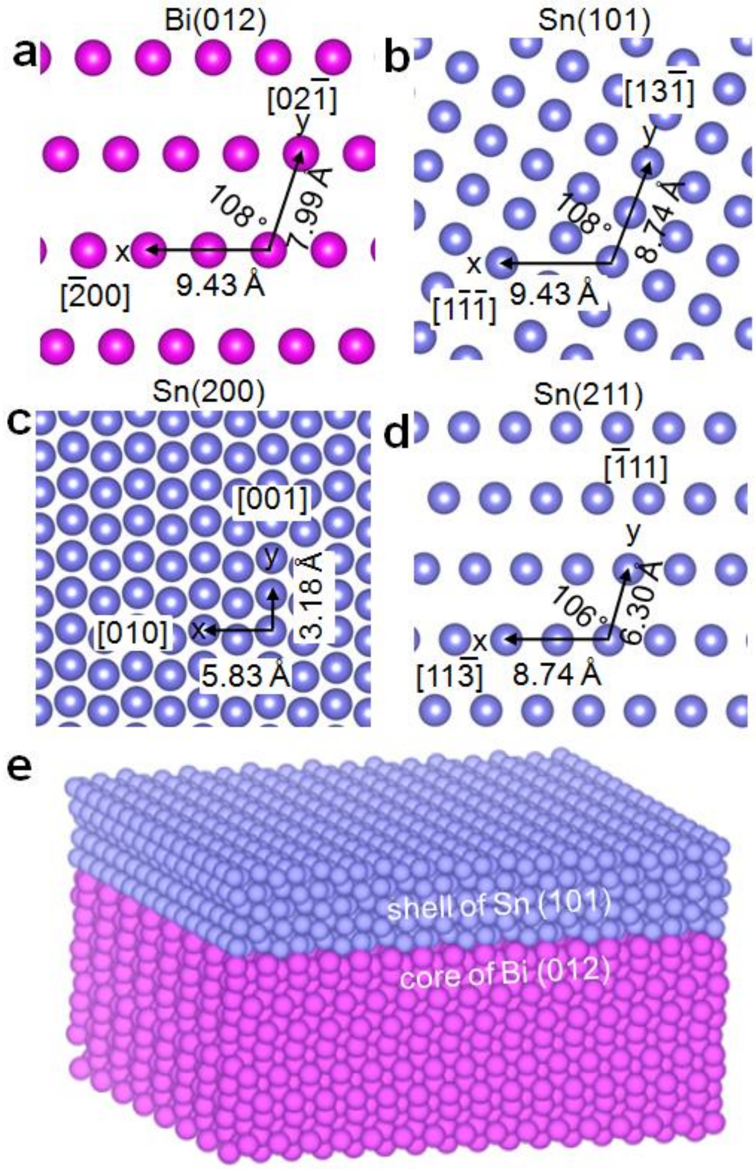

Figure S18. The lattice constants of (a) Bi (012), (b) Sn (101), (c) Sn (200), and (d) Sn (211) planes. (e) Schematic diagram of the Sn/Bi interface.

Table S2. Gibbs free energy and related correction parameters for gas phase molecules.

Species	E_{DFT} (eV)	*ZPVE* (eV)	*TS* (eV)	*G* (eV)
H_2O	-14.22	0.57	-0.34	-13.99
CO_2	-22.99	0.31	-0.66	-23.34
H_2	-6.76	0.27	-0.40	-6.89
CO	-14.80	0.14	-0.61	-15.27
HCOOH	-29.75	0.89	-0.40	-29.26

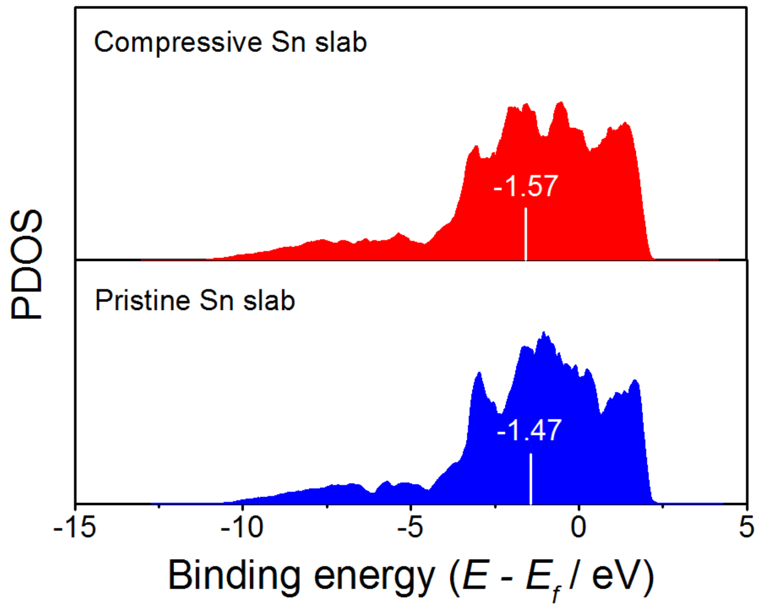

Figure S19. PDOS of surface atoms on compressive Sn slab and pristine Sn slab.

Table S3. The bond length between adsorbed species and the reaction sites of compressive Sn slab and pristine Sn slab.

	***HCOO (Sn-O)**	***COOH (Sn-C)**	***H (Sn-H)**
**pristine Sn slab**	2.203 Å	2.313 Å	1.769 Å
**compressive Sn slab**	2.221 Å	2.319 Å	1.781 Å

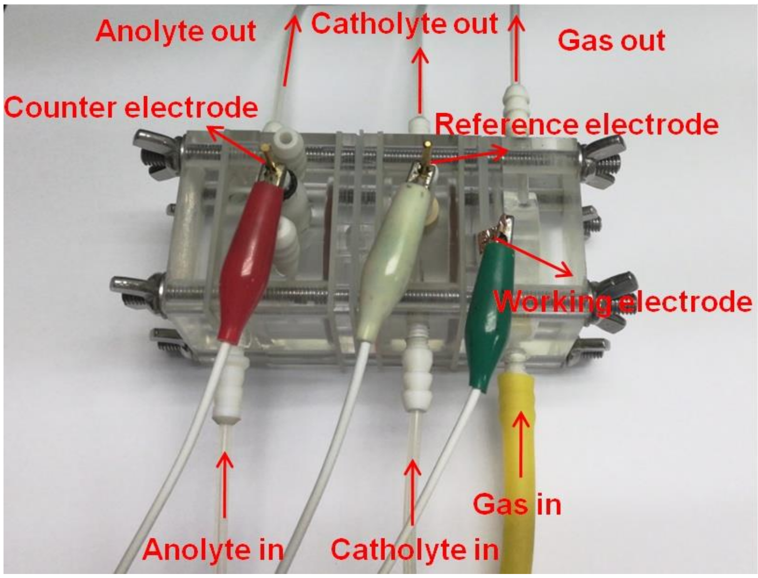

Figure S20. Photograph of the flow-cell system in this work.

Table S4. Comparison of the catalytic performance for Bi@Sn with recently reported Sn-based catalysts.

Catalysts	Potential (V *vs* RHE)	Electrolyte	j_{HCOOH} (mA cm^{-2})	FE_{HCOOH} (%)	Ref
**nano-SnO_2/graphene**	-1.15	0.1 M $NaHCO_3$	9.5	93.6	[S4]
**SnO_2 nanowire**	-1.00	0.1 M $KHCO_3$	7.8	78	[S5]
**1D SnO_2**	-0.99	0.1 M $KHCO_3$	4.7	63	[S6]
**Sn/SnS_2**	-0.75	0.5 M $NaHCO_3$	11.7	84.5	[S7]
**Sn dendrite**	-1.36	0.1 M $KHCO_3$	12.2	71.6	[S8]
**Sn GDE**	-1.20	0.5 M $KHCO_3$	17.4	78.6	[S9]
**SnO_2**	-0.60	0.5 M NaOH	2.4	67.6	[S10]
**Sn-CF1000**	-0.89	0.5 M $KHCO_3$	11.0	62	[S11]
**SnO_2 quantum wires**	-1.156	0.1 M $KHCO_3$	13.7	87.3	[S12]
**mesoporous SnO_2**	-1.15	0.1 M $KHCO_3$	8.1	75	[S13]
**5 nm SnO_2/C**	-0.9	0.1 M $KHCO_3$	4.0	54.2	[S14]
**ultrasmall SnO_2/graphene**	-0.8	0.5 M $NaHCO_3$	25.0	78	[S15]
**FSP-SnO_2**	-1.1	0.1 M $KHCO_3$	20.1	85	[S16]
**SnO_x nanosheet**	~ -1.25	0.5 M $KHCO_3$	8.3	77	[S17]
**ultrasmall SnO_2/GDE**	-1.21<br>-0.95	1.0 M $KHCO_3$<br>1.0 M KOH	92.8<br>67.6	64<br>46	[S18]
**Sn/GDE**	~ -0.9	0.45 M $KHCO_3$<br>+ 0.5 M KCl	111.2	55.6	[S19]
**electrodeposited Sn/GDE**	-1.1	0.5 M $NaHCO_3$	~ 9.2	71	[S20]
**Bi@Sn NPs**	-1.1	0.5 M $KHCO_3$	31.0	91.0	This work
**Bi@Sn NPs/GDE**	~ -1.15	2.0 M $KHCO_3$	230.5	92.2	This work

References

[S1] G. Kresse, J. Furthmüller, *Phys. Rev. B* **1996**, *54*, 11169.

[S2] J. P. Perdew, K. Burke, M. Ernzerhof, *Phys. Rev. Lett.* **1996**, *77*, 3865.

[S3] P. E. Blöchl, *Phys. Rev. B* **1994**, *50*, 17953.

[S4] S. Zhang, P. Kang, T. J. Meyer, *J. Am. Chem. Soc.* **2014**, *136*, 1734.

[S5] B. Kumar, V. Atla, J. P. Brian, S. Kumari, T. Q. Nguyen, M. Sunkara, J. M. Spurgeon, *Angew. Chem. Int. Ed.* **2017**, *56*, 3645.

[S6] L. Fan, Z. Xia, M. Xu, Y. Lu, Z. Li, *Adv. Funct. Mater.* **2018**, *28*, 1706289.

[S7] F. Li, L. Chen, M. Xue, T. Williams, Y. Zhang, D. R. MacFarlane, J. Zhang, *Nano Energy* **2017**, *31*, 270.

[S8] D. H. Won, C. H. Choi, J. Chung, M. W. Chung, E. H. Kim, S. I. Woo, *ChemSusChem* **2015**, *8*, 3092.

[S9] Q. Wang, H. Dong, H. Yu, *J. Power Sources* **2014**, *271*, 278.

[S10] S. Lee, J. D. Ocon, Y.-I. Son, J. Lee, *J. Phys. Chem. C* **2015**, *119*, 4884.

[S11] Y. Zhao, J. Liang, C. Wang, J. Ma, G. G. Wallace, *Adv. Energy Mater.* **2018**, *8*, 1702524.

[S12] S. Liu, J. Xiao, X. F. Lu, J. Wang, X. Wang, X. W. Lou, *Angew. Chem. Int. Ed.* **2019**, *58*, 8499.

[S13] R. Daiyan, X. Lu, W. H. Saputera, Y. H. Ng, R. Amal, *ACS Sustainable Chem. Eng.* **2018**, *6*, 1670.

[S14] Yiliguma, Z. Wang, C. Yang, A. Guan, L. Shang, A. M. Al-Enizi, L. Zhang, G. Zheng, *J. Mater. Chem. A* **2018**, *6*, 20121.

[S15] B. Zhang , Z. Guo, Z. Zuo, W. Pan, J. Zhang, *Appl. Catal. B-Environ.* **2018**, *239*, 441-449.

[S16] R. Daiyan, E. C. Lovell, N. M. Bedford, W. H. Saputera, K.-H. Wu, S. Lim, J. Horlyck, Y. H. Ng, X. Lu, R. Amal, *Adv. Sci.* **2019**, 6, 1900678.

[S17] Q. Zhang, Y. Zhang, J. Mao, J. Liu, Y. Zhou, D. Guay, J. Qiao, *ChemSusChem* **2019**, *12*, 1443.

[S18] C. Liang, B. Kim, S. Yang, Y. L. Yang Liu, C. Francisco Woellner, Z. Li, R. Vajtai, W. Yang, J. Wu, P. J. A. Kenis, P. M. Ajayan, *J. Mater. Chem. A* **2018**, *6*, 10313.

[S19] A. Del Castillo, M. Alvarez-Guerra, J. Solla-Gullón, A. Sáez, V. Montiel, A. Irabien, *J. CO_2 Util.* **2017**, *18*, 222.

[S20] E. Irtem, T. Andreu, A. Parra, M. D. Hernández-Alonso, S. García-Rodríguez, J. M. Riesco-García, G. Penelas-Pérez, J. R. Morante, *J. Mater. Chem. A* **2016**, *4*, 13582.
